# Genetic analysis of ectopic growth suppression during planar growth of integuments mediated by the Arabidopsis AGC protein kinase UNICORN

**DOI:** 10.1186/1471-2229-13-2

**Published:** 2013-01-02

**Authors:** Balaji Enugutti, Kay Schneitz

**Affiliations:** 1 Present address: Institute of Molecular Biotechnology of the Austrian Academy of Sciences (IMBA), Dr. Bohr-Gasse 3-5, 1030, Vienna, Austria; 2 Entwicklungsbiologie der Pflanzen, Wissenschaftszentrum Weihenstephan, Technische Universität München, Emil-Ramann-Strasse 4, 85354, Freising, Germany

**Keywords:** Arabidopsis, Auxin, ABERRANT TESTA SHAPE, AGC protein kinase, AUXIN RESPONSE FACTOR 4, Cell division, Cytokinin, ETTIN, Growth regulation, KANADI, Planar growth, Ovule, Signal transduction, UNICORN

## Abstract

**Background:**

The coordination of growth within a tissue layer is of critical importance for tissue morphogenesis. For example, cells within the epidermis undergo stereotypic cell divisions that are oriented along the plane of the layer (planar growth), thereby propagating the layered epidermal structure. Little is known about the developmental control that regulates such planar growth in plants. Recent evidence suggested that the Arabidopsis AGC VIII protein kinase UNICORN (UCN) maintains planar growth by suppressing the formation of ectopic multicellular protrusions in several floral tissues including integuments. In the current model UCN controls this process during integument development by directly interacting with the ABERRANT TESTA SHAPE (ATS) protein, a member of the KANADI (KAN) family of transcription factors, thereby repressing its activity. Here we report on the further characterization of the UCN mechanism.

**Results:**

Phenotypic analysis of flowers of *ucn-1* plants impaired in floral homeotic gene activity revealed that any of the four floral whorls could produce organs carrying *ucn-1* protrusions. The ectopic outgrowths of *ucn* integuments did not accumulate detectable signals of the auxin and cytokinin reporters *DR5rev::GFP* and *ARR5::GUS*, respectively. Furthermore, wild-type and *ucn-1* seedlings showed similarly strong callus formation upon in vitro culture on callus-inducing medium. We also show that ovules of *ucn-1* plants carrying the dominant *ats* allele *sk21-D* exhibited more pronounced protrusion formation. Finally ovules of *ucn-1 ett-1* double mutants and *ucn-1 ett-1 arf4-1* triple mutants displayed an additive phenotype.

**Conclusions:**

These data deepen the molecular insight into the *UCN*-mediated control of planar growth during integument development. The presented evidence indicates that *UCN* downstream signaling does not involve the control of auxin or cytokinin homeostasis. The results also reveal that UCN interacts with ATS independently of an ATS/ETT complex required for integument initiation and they further emphasize the necessity to balance UCN and ATS proteins during maintenance of planar growth in integuments.

## Background

In plant tissue morphogenesis the control of cell division patterns is crucial for the establishment and propagation of tissue layers. Spatially restricted asymmetric cell divisions frequently generate new cell layers. Subsequently, symmetric cell divisions maintain a cell layer, often by aligning the division planes along the plane of the layer (planar growth). The regulation of asymmetric cell divisions is under intense scrutiny [1-5]. By contrast, the developmental control of planar growth is largely unknown [6].

There is evidence for a link between the control of adaxial-abaxial polarity and the laminar growth of the leaf blade. Leaves are lateral determinate organs and are characterized by a distinct adaxial-abaxial or dorsal-ventral polarity across the whole multi-layered organ. Outgrowth of the developing leaf lamina is believed to require stimulation of cells located at the adaxial-abaxial boundary [7]. The control of adaxial-abaxial leaf polarity relies on the antagonistic interactions between Class III HD-ZIP and KANADI (KAN) transcription factors [8,9]. Class III HD-ZIP genes promote adaxial identity [10-13] and *KAN* genes, in conjunction with auxin response factor genes *ETTIN* (*ETT*) and *ARF4*, direct abaxial cell fate and lamina outgrowth in leaves [10,14-16]. Interestingly, defects in the control of adaxial identity can result in localized ectopic blade-like outgrowths on adaxial surface of affected leaves [13,17]. Similarly, it has been observed that misregulation of abaxial leaf polarity can lead to ectopic blade-like outgrowths on the abaxial side of leaves and cotyledons [18,19].

Evidence for a connection between the regulation of adaxial-abaxial polarity and planar growth also comes from studies using Arabidopsis integuments as model system [20,21]. Integuments are lateral determinate tissues of ovules and the progenitors of the seed coat. Arabidopsis ovules develop an inner and outer integument of entirely epidermal origin [22,23]. Upon initiation they form laminar extensions of distinct adaxial-abaxial polarity each consisting of two cell layers of anticlinally dividing cells. The outer integument will grow asymmetrically and eventually envelop the inner integument and the developing embryo sac.

Recently it was discovered that maintenance of planar growth of integuments is under control of *UNICORN* (*UCN*) [20,21]. *UCN* encodes a functional protein kinase that belongs to the AGC2 subclass of the plant-specific AGC VIII family [24-27]. Integuments of recessive *ucn* mutants exhibit local disorganized growth resulting in the formation of one to several multicellular protrusions containing cells with at least partial integument identity. Similar protrusions are also present on stamens and petals. At the cellular level, the earliest detectable defects are local periclinal or oblique cell divisions in individual cell layers. They are clearly distinct from the typical anticlinal cell divisions that maintain planar growth of integuments. In addition, *ucn* proembryos show altered cell division planes and double mutants carrying null alleles of *UCN* and its closest homolog *UNICORN-LIKE* (*UCNL*) are embryo lethal. These observations indicated that *UCN* suppresses ectopic growth by influencing division planes in symmetrically dividing cells.

*UCN* maintains planar growth during integument outgrowth by interacting with *ABERRANT TESTA SHAPE* (*ATS*) [21]. *ATS* is a *KAN* gene required for several processes of integument development including, integument boundary formation, inner integument outgrowth and the control of adaxial-abaxial polarity [28-31]. In addition, the ATS protein appears to form a functional complex with the auxin response factor ETTIN (ETT) [32] to control early integument development [33]. Protrusion formation in integuments of *ucn ats* double mutants is strongly diminished indicating that *UCN* represses *ATS*[21]. This negative regulation is likely to occur through physical interaction of the two proteins as *ATS* transcript levels are unaltered in *ucn* mutants and recombinant UCN protein is able to phosphorylate ATS in in vitro kinase assays. Moreover, bimolecular fluorescence complementation (BiFC) analysis further supports direct physical interaction between UCN and ATS [21]. Thus, by inhibiting ATS UCN appears to prevent misregulation of transcriptional programs that control planar growth in integuments.

Here we further characterize *UCN*-mediated maintenance of planar growth during integument development. We provide evidence that *UCN* functions in an organ-specific manner and that *UCN* does not influence auxin and cytokinin homeostasis. Our data further suggest that UCN and ATS protein levels must be balanced and that repression of ATS by UCN does not involve either ETT or the ATS/ETT complex.

## Results and discussion

### *UCN* functions in an organ- not a whorl-specific manner in floral organogenesis

**Figure 1:**
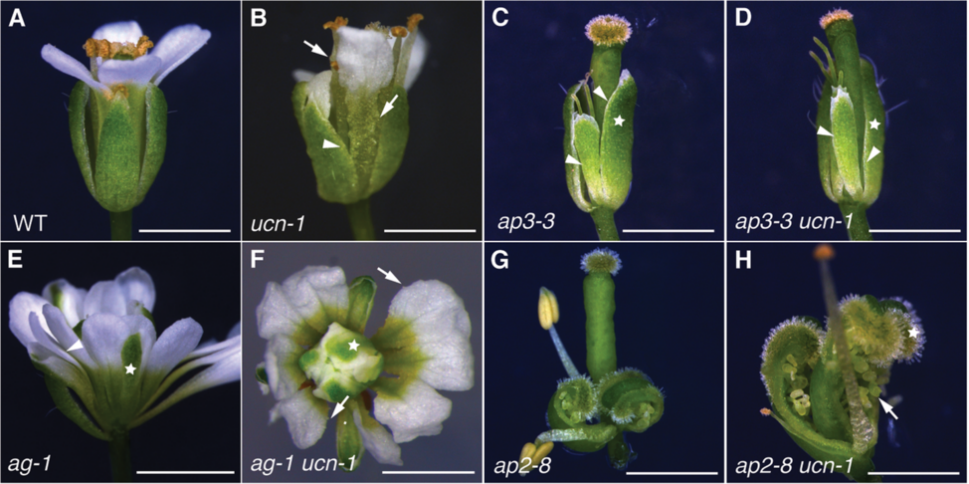
**Phenotypic analysis of *ap3 ucn*, *ag ucn *and *ap2 ucn *flowers.** Light micrographs of stage 14 flowers. Arrows indicate protrusions, arrowheads indicate absence of protrusions. (**A**) Wild-type (L*er*). (**B**) *ucn-1*. Note the presence of protrusions on petals and stamens but their absence on sepals. (**C**) *ap3-3*. One first-whorl sepal was removed to reveal the inner second-whorl sepal. The star indicates a first-whorl sepal. (**D**) *ap3-3 ucn-1* double mutant. Similar setup as in (**C**). Note the absence of protrusions on the second-whorl sepal. Serrations at the tip of the second-whorl sepal regularly occur in *ap3-3* single mutants. (**E**) *ag-1* flower. Star indicates a fourth-whorl sepal. (**F**) *ag-1 ucn-1* double mutant. Protrusions are found on second- and third-whorl petals. (**G**) *ap2-8*. First-whorl unfused carpels with ovules are visible. (**H**) *ap2-8 ucn-1*. The first-whorl ovules carry protrusions. Scale bars: 0.5 mm.

**Figure 2:**
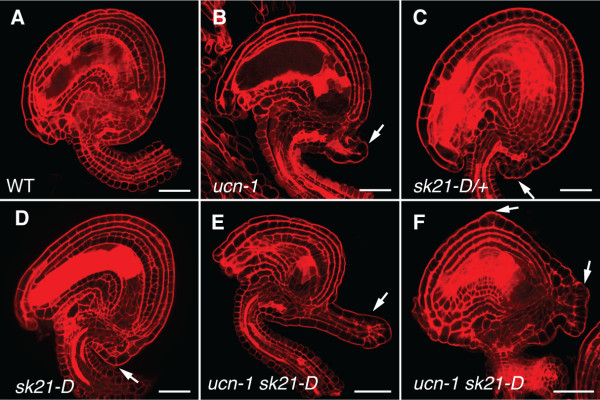
**Ovules of *ucn-1 sk21-D *mutants show enhanced protrusion formation.** Confocal micrographs of early stage 4 ovules stained with pseudo-Schiff propidium iodide (mPS PI) are shown. Arrows indicate protrusions. (**A**) Wild-type ovule (L*er*). (**B**) *ucn-1* ovule. Note the presence of a protrusion. (**C**) *sk21-D/+* ovule. A small protrusion is indicated. (**D**) *sk21-D* ovule. (**E**, **F**) *ucn-1 sk21-D* ovules. (**E**) Note the prominently enlarged protrusion (compare with **B**, **D**). (**F**) Multiple protrusions are detectable. Scale bars: 20 μm.

Flowers carry four different types of floral organs arranged in whorls. In Arabidopsis, sepals occupy whorl 1, petals whorl 2, stamens whorl 3 and carpels including the ovules whorl 4 [34]. According to the ABC model floral organ identity is specified at the whorl level by a set of floral homeotic genes, encoding mostly MADS-domain transcription factors, that act in a combinatorial fashion [35-37]. Interestingly, *ucn* mutants show protrusions in petals, stamen and ovules (Figure 1B, Figure 2B) [21], however, we never observed protrusions on sepals or carpels. This observation raised the question whether *UCN* acts in an organ- or whorl-specific manner.

To address this issue we generated a set of double mutants between *ucn-1* and several floral homeotic mutants (Figure 1). Flowers of *apetala3* (*ap3*) mutants carry sepals in whorl 2 and carpels in whorl 3 [38]. The second whorl sepals of *ucn-1 ap3-3* flowers did not show *ucn*-like protrusions (Figure 1D), although the second-whorl petals of *ucn-1* mutants do, providing first evidence that *UCN* acts in an organ and not a whorl-specific manner. To test this assumption further we analyzed two additional combinations. Plants with a defect in *AGAMOUS* (*AG*) exhibit petals in the third whorl and an additional flower in the fourth whorl [38]. Third-whorl petals of *ucn-1 ag-1* still showed protrusions (Figure 1F). In *apetala2* (*ap2*) mutants unfused carpels with ovules and stamens develop in the first whorl and second whorl, respectively [38-40]. Flowers of *ucn-1 ap2-8* double mutants were characterized by first-whorl carpels devoid of protrusions but that included *ucn*-like ovules (Figure 1H). Thus, in particular the phenotypes of *ucn-1 ap3-3* and *ucn-1 ap2-8* flowers suggest that *UCN* acts in an organ-specific rather than whorl-specific manner in floral organogenesis.

### Outgrowths in *ucn-1* integuments develop autonomously of auxin and cytokinin

The maintenance of plant tissue morphogenesis and the prevention of aberrant growth and tumor formation is under hormonal and genetic control [41-43]. For example, callus formation can be induced at non-wounding sites in explants by in vitro auxin and cytokinin treatment [44,45] resulting in masses of partially dedifferentiated cells that resemble root meristem tips [46-48]. Furthermore, defects in *PROPORZ1* (*PRZ1*), encoding a putative component of a chromatin-remodeling complex, result in callus formation upon addition of auxin or cytokinin [49]. In addition, ectopic expression of *AINTEGUMENTA* (*ANT*), encoding an AP2-class transcription factor involved in the control of cell division and organ initiation [50-57], results in unorganized cell proliferation in wounded or detached ends of fully differentiated leaves [58]. *ANT* acts downstream of auxin and regulates meristematic competence during organogenesis [58,59].

**Figure 3:**
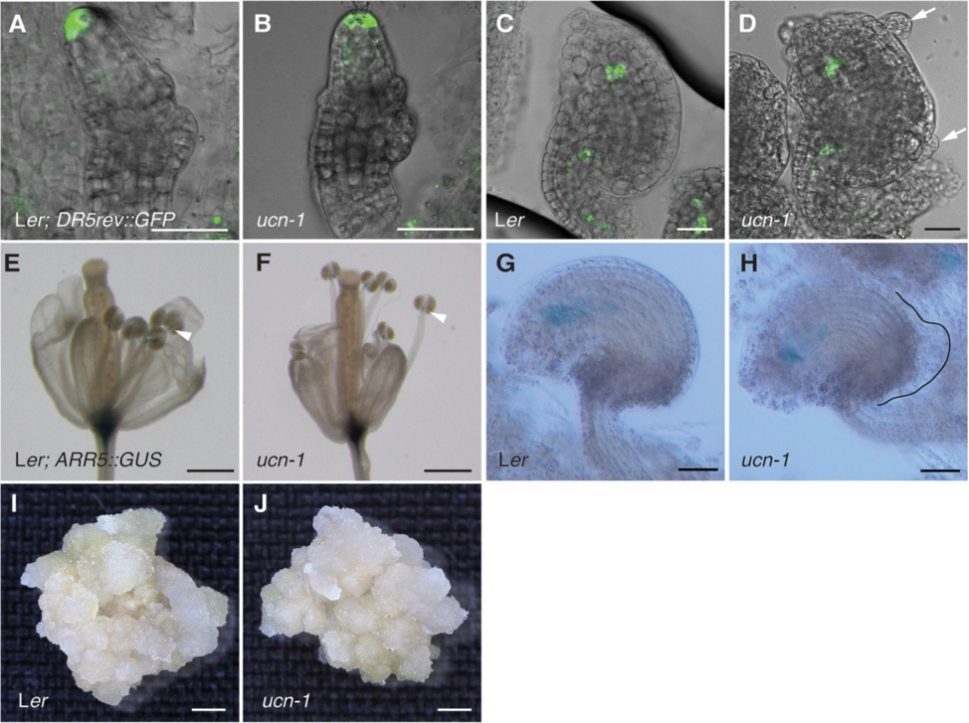
**Hormone-induced callus formation is not affected in *ucn-1 *seedlings and *ucn-1 *protrusions do not accumulate detectable *DR5rev::GFP *and *ARR5::GUS *reporter signals.** (**A-D**) Confocal micrographs of live wild-type (L*er*) and *ucn-1* plants carrying the *DR5rev::GFP* reporter. Overlays of GFP and bright-field channels. No difference in signal detected between wild-type and *ucn-1*. (**A, B**) Stage 2-III ovules. (**C, D**) Stage 3-III ovules. Note the absence of detectable signal in the *ucn-1* protrusions (arrows). (**E-H**) Plants carrying the *ARR5::GUS* reporter. No difference in signal detected between wild-type and *ucn-1*. (**E**, **F**) Light micrographs of fixed stage 14 flowers (4 h incubation in GUS staining solution). Arrowheads indicate signal at the junction between filament and anther. (**G**, **H**) Differential interference contrast micrographs of fixed stage 3-IV ovules (16 h incubation in GUS staining solution). Note the absence of signal in the *ucn-1* protrusion (outlined). (**I**) Light micrograph of a 30-days-old callus grown from germinated wild-type (L*er*) seedling grown on callus induction medium (CIM). (**J**) Light micrograph of a 30-days-old callus grown from germinated *ucn-1* seedling grown on CIM. Callus is of comparable size than the one in (**I**). Scale bars: (**A-D**): 20 μm, (**G**, **H**) 30 μm, (**E**, **F**, **I**, **J**) 0.5 mm.

To explore the relationship between *UCN* and auxin as well as cytokinin we tested whether the integument protrusions of *ucn-1* expressed the well-characterized reporters *DR5rev::GFP* or *ARR5::GUS* that act as proxies for the presence of auxin and cytokinin, respectively [60,61]. We observed *DR5rev::GFP* signal distribution as reported previously in developing ovules, such as the tip of the ovule primordium or the micropylar end of the young embryo sac [61,62]. Interestingly, however, no signal could be seen in variably advanced protrusions of *ucn-1* integuments (Figure 3A-D). *ARR5::GUS* expression could be observed in the tip of filaments as noted earlier [63]. During ovule development we could also detect a signal in the developing embryo sac. The latter signal is in accordance with the expression pattern of the *IPT1::GUS* reporter, using the promoter of a cytokinin biosynthesis enzyme [64,65], and the synthetic cytokinin reporter *TCSpro::GFP*[65,66]. However, we did not observe *ARR5::GUS* signal in developing protrusions of *ucn-1* integuments (Figure 3E-H). These results suggest that *ucn-1* integument protrusions do not accumulate auxin or cytokinin, at least not to a level or in a fashion detectable by these two reporters.

To assess further the relationship between *UCN* and auxin as well as cytokinin we tested whether *UCN* influences callus formation in seedlings treated with exogenous auxin and cytokinin. However, when comparing wild-type and *ucn-1* seedlings grown on callus inducing medium (CIM) we detected no difference in size or number of formed calli (Figure 3I, J) (n = 25). Thus, it appears that *UCN* does not affect hormone-induced callus formation in this assay.

The reporter-based experiments described above need to be interpreted with caution. Still, the results are also in accordance with previous genetic data. For example, the additive ovule phenotype of *ucn bel1* double mutants [21] further supports the notion that *ucn* integument protrusions develop autonomously of auxin. *BEL1* encodes a homeodomain transcription factor required for chalaza development [20,67-69]. Plants lacking *BEL1* activity develop ovules carrying large protrusions emanating from the chalaza. Interestingly, young protrusions of *bel1* ovules show ectopic expression of a *PIN1::PIN1:GFP* reporter [65], used to assess the presence of the polar auxin transport facilitator PIN1 [70,71]. Furthermore, *bel1* mutants treated with the polar auxin transport inhibitor N-1-naphthylphthalamic acid (NPA) failed to form protrusions. These results indicate that auxin contributes to the formation of *bel1* outgrowths [65]. If auxin would play a major role in the induction of outgrowth formation in *ucn-1* integuments one might expect enlarged protrusion formation in *ucn bel1* double mutants. However, this is not the case, as *ucn bel1* double mutants did not show a noticeable increase in protrusion size [21]. In addition, protrusion formation still occurs on petals and stamen in *ucn ant* double mutants [21].

Available evidence suggests that cytokinin may be of little importance for the control of integument outgrowth. Signaling mediated by the three cytokinin receptor genes *CRE1*, *AHK2*, and *AHK3*[72] appears to be critical for ovule primordium outgrowth [65] and embryo sac development [65,73]. However, these genes appear to play a minor role if any during integument outgrowth as either no defects in integument development [73] or only a frequency of 10 percent of finger-like ovules lacking integuments [65] were reported in strong *cre1 ahk2 ahk3* triple mutants. These affected ovules may have still suffered from defects occurring during prior primordium outgrowth. The absence of detectable *ARR5::GUS* expression in *ucn-1* protrusions, consisting of at least partially differentiated integument cells, may thus reflect the minor role of cytokinin in integument outgrowth.

Taken together the available evidence suggests that processes functioning downstream of *UCN* growth suppression do not involve the regulation of auxin and cytokinin homeostasis.

### Relative levels of UCN and ATS are critical for planar growth of integuments

The current model states that UCN maintains planar growth in integuments by directly repressing the activity of the ATS protein implying that the balance between UCN and ATS proteins may be crucial in this process. Previously we could show that an about 45-fold increase of *ATS* transcript levels within its normal spatial expression domain in the activation tagging mutant *sk21-D*[74] is accompanied by *ucn*-like protrusion formation in integuments [21]. This result supports the genetic model that *UCN* is a negative regulator of *ATS*. One interpretation of the *sk21-D* integument phenotype includes the assumption that elevated *ATS* transcript levels lead to higher than normal amounts of ATS protein, which may titrate out available UCN. If this notion is correct one might expect that upon reduction of *UCN* function even more exaggerated protrusion formation should take place in *sk21-D* plants.

To test this notion we performed a genetic gene dosage assay (Figure 2, Table 1). Ovules of *ucn-1/+* plants do not show protrusions [21]. Analysis of ovules of *sk21-D*/+ heterozygous plants revealed less prominent protrusion formation than in ovules of *sk21-D* homozygous plants (Figure 2C, D). Furthermore, we generated *ucn-1* plants that were either heterozygous or homozygous for *sk21-D*. Indeed ovules of these mutants showed an increase in protrusion formation, both in terms of protrusion size (Figure 2E) and in number of protrusions formed (Figure 2F, Table 1). Strongest effects were seen in *ucn-1 sk21-D* double mutants. A dosage effect was discernable as *ucn-1* plants heterozygous for *sk21-D* showed an intermediate phenotype compared to *ucn-1* or *ucn-1* homozygous for *sk21-D* (Table 1).

**Table 1:** **Quantification of protrusion number on ovules of wild-type, *ucn-1*, *sk21-D*, and *ucn-1 sk21-D *mutants**

**Genotype**	**No. of ovules analyzed**	**No. of ovules with only one protrusion**	**No. of ovules with *>*1 protrusions**	**% of ovules with protrusions**
WT	200	0	0	0
*ucn-1*	200	96	89	92.5
*sk21-D/+*	200	11	0	5.5
*sk21-D*	200	87	2	44.5
*ucn-1 sk21-D/+*	200	93	95	94
*ucn-1 sk21-D*	200	34	158	96

Taken together these data support the model that elevated transcript levels of *ATS* can ultimately lead to out-titration of functional UCN. In addition, they provide further genetic evidence that it is indeed critical to maintain a proper balance between UCN and ATS protein levels in the regulation of planar integument growth.

### *UCN* acts independently of *ETT* and *ARF4* during integument development

Genetic studies indicated that *AUXIN RESPONSE FACTOR* (*ARF*) gene *ETTIN* (*ETT*) and its closest homolog *ARF4* are required for the control of adaxial-abaxial leaf polarity in conjunction with *KAN1* and *KAN2*[16]. Furthermore, the ETT and ATS proteins may physically interact to form a functional complex required for integument development and polarity [33]. Our previous data suggested that UCN maintains planar growth of integuments by negatively regulating the polarity factor ATS through a physical interaction between the two proteins [21]. If interaction between ATS and ETT proteins is crucial for the formation of a functional complex then impairing either protein should result in similar absence of function. As a consequence *ats* and *ett* should show a similar genetic behavior with respect to *ucn*.

**Figure 4:**
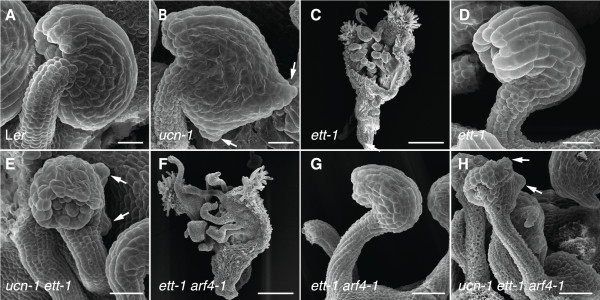
**Ovules of *ucn-1 ett-1 *and *ucn-1 ett-1 arf4-1 *mutants show additive phenotypes.** Scanning electron micrographs of early stage 4 ovules are depicted with the exception of (**C**, **F**) which show gynoecia of stage 14 flowers. (**A**) Wild-type ovule. (**B**) *ucn-1* ovule. Arrows highlight protrusions. (**C**) Typical gynoecium of an *ett-1* flower. (**D**) *ett-1* ovule. (**E**) Additive phenotype of a *ucn-1 ett-1* double mutant ovule. Note the protrusions (arrows). (**F**) Typical gynoecium of an *ett-1 arf4-1* flower. (**G**) *ett-1 arf4-1* ovule. Not noticeably different from an *ett-1* ovule (compare with **D**). (**H**) Additive phenotype of a *ucn-1 ett-1 arf4-1* triple mutant ovule. Not noticeably different from a *ucn-1 ett-1* double mutant ovule (compare with **E**). Arrows mark protrusions. Scale bars: 20 μm.

To test this hypothesis we generated *ucn-1 ett-1* double and *ucn-1 ett-1 arf4-1* triple mutants and analyzed their ovule phenotypes (Figure 4). In agreement with a repressive role of UCN on ATS activity *ats* is epistatic to *ucn-1* in *ucn-1 ats-3* double mutants [21]. Ovules of *ett-1* mutants displayed an *ats*-like phenotype (Figure 4D) confirming earlier results [33]. Surprisingly, however, ovules of *ucn-1 ett-1* double mutants (Figure 4D) or *ucn-1 ett-1 arf4-1* triple mutants (Figure 4H) exhibited an additive phenotype. These results suggest that *UCN* and *ETT/ARF4* function in different pathways. They further indicate that *UCN* does not participate in auxin-related aspects of integument development that involve these two auxin response factors.

Interestingly, the additive ovule phenotype of *ucn-1 ett-1* double mutants and *ucn-1 ett-1 arf4-1* triple mutants seems not in accordance with the notion of a functional ATS/ETT complex. How can this apparent discrepancy be resolved? At least two models are conceivable. In one scenario the ATS/ETT complex is required throughout integument development and UCN inactivates ATS that is not in a complex with ETT. Alternatively, the ATS/ETT complex is transient and only active during early integument development. Sometime after integument initiation the ATS/ETT complex ceases to be required and dissociates. UCN then interacts with now available free ATS and represses its activity. We favor the latter model as the *ATS*-dependent protrusions in integuments of *ucn-1* or *sk21-D* mutants first appear once integuments are initiated and continue to outgrow [21].

Both models detailed above imply that ATS protein that is not bound to ETT interferes with the transcriptional programs regulating planar integument growth and must be repressed. Thus, the new genetic data presented above are in agreement with and at the same time refine our current view on the UCN-mediated control of planar integument growth.

## Conclusions

Here we provide genetic data that *UCN* acts in an organ-specific manner during floral development. Furthermore, the repression of ectopic growth by *UCN*-mediated signaling does not appear to involve a control of auxin or cytokinin homeostasis. *UCN*-dependent growth suppression is mediated through a repression of ATS and requires a critical balance of both proteins. This repression is independent of a likely earlier-acting ATS/ETT protein complex involved in integument initiation. The presented evidence deepens our understanding of *UCN*-mediated suppression of ectopic growth. It furthers the link between the control of adaxial-abaxial polarity and planar growth and contributes to a solid experimental and conceptual foundation for further exploration of planar growth control during integument development.

## Methods

### Plant work

*Arabidopsis thaliana* (L.) Heynh. var. Columbia (Col-0) and var. Landsberg (*erecta* mutant) (L*er*) were used as wild-type strains. Plants were grown essentially as described previously [20]. The following mutants were used: *ag-1*[38,75]; *ap2-8*[35], *ap3-3*[76], *arf4-1*[16,77,78], *ett-1*[16,79], *sk21-D*[74], *ucn-1*[20,21]. In double-mutant studies respective double mutants were identified based on their phenotypes segregating in a Mendelian fashion. The *ucn-1 sk21-D/+* or *ucn-1 sk21-D/sk21-D* plants were genotyped using primers sk21-D (GT)_F :GAGAATTAGTACAATGTAATG, sk21-D (GT)_R :GTGATTTAACCCTTCTCAAGTGC and pSKI (GT)_F: CCACCCACGAGGAACATCGTG.

### In vitro culture and hormone treatment

Seedlings were grown in a growth room under constant light conditions at 23°C. For induction of callus freshly germinated seedlings were grown on MS plates for 6 days. Seedlings were then transferred onto MS plates supplemented with 3 μg/ml each of the auxin 1-naphthalene acetic acid (NAA) (Sigma) and the cytokinin kinetin (Sigma) (callus induction medium, CIM).

### Microscopy and art work

Preparation and analysis of samples for light microscopy, scanning electron microscopy, and histochemical localization of β-glucuronidase (GUS) activity in whole-mount tissue was done essentially as described [20,80]. Ovule staining with pseudo-Schiff propidium iodide (mPS-PI) was done as described [81]. Confocal laser scanning microscopy was performed with an Olympus FV1000 setup and FluoView software (Olympus Europa GmbH, Hamburg, Germany) as described previously [21]. Images were adjusted for color and contrast using Adobe Photoshop CS5 (Adobe, San Jose, CA, USA) software.

## Authors’ contributions

BE conceived of the study, participated in the design, and carried out experiments. KS conceived of the study, participated in the design and coordination and wrote the manuscript. All authors read and approved the final manuscript.
